# Enhancing motor skill acquisition and autonomous motivation in university physical education: a randomized controlled trial of a STEAM-integrated approach

**DOI:** 10.3389/fpsyg.2026.1754141

**Published:** 2026-05-01

**Authors:** Daoqing Zhang

**Affiliations:** School of Physical Education, Hubei University of Arts and Science, Xiangyang, China

**Keywords:** autonomous motivation, motor learning, randomized controlled trial, smartphone-based feedback, STEAM education, university physical education

## Abstract

University physical education (PE) is increasingly expected to foster physical literacy by integrating movement competence with cognitive understanding and intrinsic motivation. However, rigorous evidence regarding the effectiveness of interdisciplinary approaches, such as STEAM (Science, Technology, Engineering, Arts, and Mathematics), remains limited in higher education settings. This study designed and evaluated a STEAM-integrated Roliball (RB) curriculum that utilizes smartphone-based kinematic feedback to support motor learning and inquiry-based practice. A section-stratified randomized controlled trial (RCT) was conducted with 70 first-year university students. Participants were allocated to either an 8-week STEAM-RB intervention or a traditional syllabus. The STEAM-RB group engaged in Kolb’s experiential learning cycle, using high-frame-rate video to analyze racket trajectories and connecting biomechanical principles to skill execution. Primary outcomes included kinematic performance (peak racket-head angular velocity via video analysis), blinded expert ratings of technique, and STEAM-specific knowledge. Secondary and exploratory outcomes assessed autonomous motivation (derived from the Chinese University Students’ Physical Activity Motivation Scale, CUSPAMS) and creative disposition. Pretest-adjusted ANCOVAs revealed that the STEAM-RB curriculum yielded superior outcomes across motor and cognitive domains. Relative to the traditional group, the intervention group showed significant improvements in kinematic performance (moderate-to-large effect) and expert ratings of technique. Notably, the intervention produced a large gain in STEAM knowledge and a small-to-moderate increase in autonomous motivation, whereas creative disposition remained stable across both groups. These findings demonstrate that embedding low-cost digital technology and STEAM inquiry into university PE can significantly enhance motor skill acquisition and cognitive engagement without compromising motivation, suggesting that smartphone-supported inquiry is a viable strategy for modernizing technique-focused PE courses.

## 1 Introduction

University physical education (PE) is increasingly expected to develop movement competence, disciplinary understanding, and motivation to be active across the lifespan (State Council of the People’s Republic of China, 2019; World Economic Forum, 2023). Yet curriculum evaluation in university PE still relies heavily on coarse skill tests, attendance, and global grades, with limited use of multi-domain frameworks that combine kinematic, cognitive, and motivational outcomes (Casey et al., 2016; Wang et al., 2023; Wang et al., 2024). This leaves many technology-enhanced and inquiry-oriented reforms supported more by aspiration than by rigorous evidence (Ntoumanis et al., 2021; Sargent and Calderón, 2022).

Digital technologies offer one route to strengthen both pedagogy and measurement. High-frame-rate smartphone video, simple motion analysis software, and accessible kinematic proxies can render movement technique visible at low cost, enabling feedback-rich instruction and research-grade assessment in authentic PE settings (Casey et al., 2016; Staacks et al., 2018; Pueo et al., 2020). Systematic reviews and meta-analyses indicate that video-based feedback and digital technology can enhance motor learning and that mobile devices can provide valid and reliable kinematic data for selected tasks (Mödinger et al., 2022; Pueo et al., 2020; Meng, 2023). However, in most university PE curricula these tools are added as short-term “extras” rather than embedded in a coherent pedagogical and measurement design (Leavy et al., 2023; Sargent and Calderón, 2022).

In parallel, STEAM (science, technology, engineering, arts, and mathematics) approaches have been proposed as a way to integrate disciplinary thinking and creative inquiry into PE and school-based physical activity programs (Perignat and Katz-Buonincontro, 2019; Tran et al., 2021; Yuan et al., 2022). STEAM-oriented PE units can ask students to formulate and test kinematic hypotheses, model trajectories, or justify equipment modifications, linking embodied practice with scientific and technological reasoning (Conradty and Bogner, 2019; Leavy et al., 2023). Emerging studies suggest that such designs can support conceptual understanding and motivation, particularly when tasks are tightly aligned with movement practice and assessment captures more than final products (Yuan et al., 2022; Rominger et al., 2022; Vasilopoulos et al., 2023; Qi et al., 2024). In higher education, however, STEAM-integrated PE units remain rare, and few have been examined using randomized designs or multi-domain outcome measures.

Roliball (RB)—a racket sport drawing on Tai Chi principles of continuous, spiral movement—provides a suitable model task for STEAM integration in university PE. RB is technically demanding yet relatively safe, amenable to high-frame-rate smartphone video capture, and already used in some Chinese university PE programs, including STEAM-oriented pilots (Yuan et al., 2022). Importantly, key technique constraints (e.g., maintaining centripetal force and controlling tangential release angles) are readily observable via simple kinematic analysis, yet remain difficult for novices to master without timely, interpretable feedback. These characteristics make RB an appropriate context for a STEAM-integrated curriculum that couples movement practice with kinematic feedback, guided inquiry, and structured reflection, while enabling rigorous evaluation across performance, knowledge, and motivational outcomes (State Council of the People’s Republic of China, 2019).

Kolb’s experiential learning cycle offers a framework for organizing such a curriculum, emphasizing iterative cycles of concrete experience, reflective observation, abstract conceptualization, and active experimentation (Kolb, 2015). Embedding smartphone-based kinematic feedback and guided inquiry within this cycle may help students connect embodied experience with scientific concepts while also yielding sensitive outcome measures. The present trial designs and evaluates a theory-grounded STEAM–Roliball (STEAM-RB) curriculum model for university PE, with a specific focus on measurement. Beyond RB, the model is intended as a transferable blueprint for modernizing technique-focused university PE units using low-cost sensing and multi-domain assessment.

**Figure 1:**
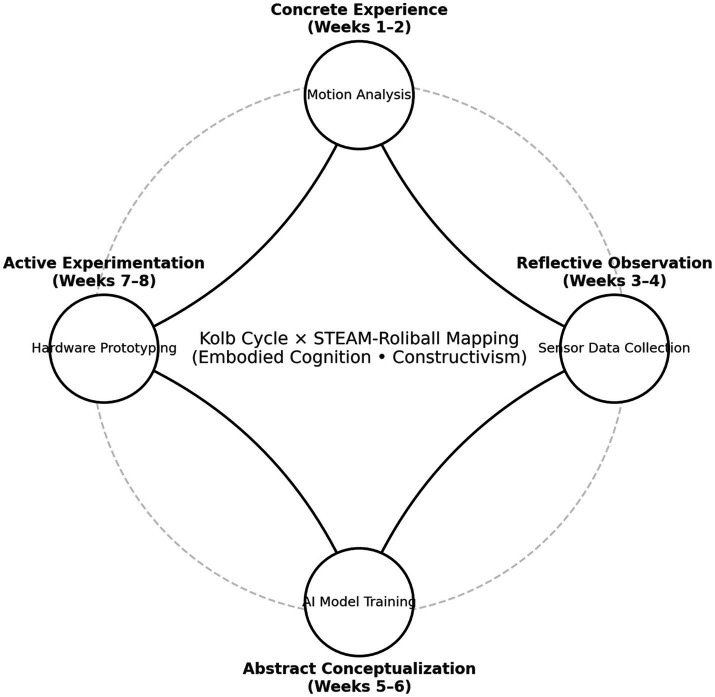
Conceptual framework linking Kolb’s experiential cycle to the STEAM–Roliball curriculum. The framework maps Kolb’s experiential learning cycle (concrete experience → reflective observation → abstract conceptualization → active experimentation) to STEAM domains (mechanics/geometry; sensing/feedback; data interpretation; engineering/aesthetics) within Roliball (RB) instruction. Low-cost tools (240 fps video, smartphone sensors, GeoGebra) provide data-driven feedback that supports motor skill acquisition and concept formation. CUSPAMS, Chinese University Students’ Physical Activity Motivation Scale; CPS, revised Creative Personality Scale; SOP = standard operating procedure.

As summarized in Figure 1, the curriculum operationalizes a Kolb-inspired experiential cycle through four linked components: (a) situated RB practice; (b) smartphone video and simple kinematic feedback; (c) STEAM-focused inquiry tasks (e.g., relating racket angle to shuttle trajectory); and (d) reflective consolidation (Casey et al., 2016; Sargent and Calderón, 2022).

While previous research has explored STEAM in general education, its application to complex motor skill acquisition in university PE remains understudied. To address this gap, this study utilizes Roliball (RB)—a racket sport characterized by continuous, circular swinging patterns—as a model system. RB provides an ideal testbed for this investigation because its technical demands (e.g., maintaining centripetal force, controlling tangent release angles) are easily visualized through smartphone-based kinematic analysis, yet difficult for novices to master without feedback.

Self-determination theory (SDT) (Deci and Ryan, 1985; Ryan and Deci, 2000) provides a complementary lens for the hypothesized motivational effects of STEAM-integrated instruction. We propose that (a) smartphone-based kinematic feedback supports perceived competence by making technique visible, interpretable, and improvable; (b) inquiry-driven STEAM tasks support autonomy by allowing students to generate questions, test ideas, and iteratively refine solutions; and (c) collaborative data interpretation supports relatedness through shared problem solving. Together, these need-supportive mechanisms are expected to promote internalization and yield larger gains in autonomous motivation (intrinsic and identified regulation) in the STEAM-RB group than in the traditional syllabus.

Therefore, the primary aim of this trial was not merely to evaluate a specific sport syllabus, but to examine the efficacy of a transferable STEAM-integrated pedagogical model. Specifically, we hypothesized that, compared to a traditional syllabus, the STEAM-integrated curriculum (incorporating smartphone video feedback and guided inquiry) would yield superior post-test outcomes in: (a) motor competence (indexed by peak racket-head angular velocity and expert ratings); (b) domain-specific cognitive understanding (STEAM knowledge); and (c) autonomous motivation for physical activity.

## 2 Methods

### 2.1 Design and participants

This single-site, section-stratified, individual-level randomized trial evaluated a STEAM-integrated Roliball (STEAM-RB) curriculum in university physical education. Two intact first-year Roliball PE sections at a public university in central China were recruited at the beginning of the semester (*N* = 70). All students were first-year undergraduates enrolled to meet a mandatory PE requirement and had no prior formal Roliball training. Participation in the research was voluntary.

### 2.2 Ethics statement

The study was conducted in accordance with the Declaration of Helsinki. At the time of the project, the host university’s policy indicated that minimal-risk, curriculum-embedded interventions in physical education did not require formal review by a central institutional review board. The protocol was reviewed and approved by the Departmental Research Committee of Hubei University of Arts and Science, which confirmed that the study met local institutional requirements for educational research. Written informed consent was obtained from all participants prior to data collection.

### 2.3 Sample size and randomization

An *a priori* power analysis (G*Power 3.1) (Faul et al., 2007) indicated that a total of 68 participants (34 per arm) would be sufficient to detect a medium effect size (*f* = 0.25) on a primary outcome using ANCOVA with one covariate (*α* = 0.05, power = 0.80). To allow for modest attrition, both intact Roliball sections were invited and all consenting students were randomized.

Within each section, students were individually randomized (1:1) to either the STEAM-RB curriculum or the traditional Roliball syllabus, stratified by section to preserve existing social and timetable structures. An independent researcher generated the random sequence and concealed allocation until assignment. This produced two mixed-composition classes in each section (STEAM-RB and traditional), with instruction delivered in parallel.

To reduce instructor effects, an instructor crossover was implemented at week 4: the two experienced Roliball instructors exchanged groups mid-intervention while keeping curriculum condition (STEAM-RB vs. traditional) unchanged. Both instructors had at least 5 years of university PE teaching experience and received joint training on the study protocol before implementation.

### 2.4 Interventions

Both groups completed two 90-min Roliball classes per week for 8 weeks (16 sessions) in standard indoor PE facilities. The traditional curriculum followed the existing university syllabus, emphasizing teacher-directed demonstration, part–whole practice of core techniques (e.g., basic swings, directional control), and summative skill testing.

The STEAM-RB curriculum adapted this syllabus within Kolb’s experiential learning cycle (concrete experience, reflective observation, abstract conceptualization, active experimentation), embedding smartphone-based kinematic feedback and STEAM-focused inquiry tasks into each unit. As summarized in Figure 1, each lesson for the STEAM-RB group involved four linked components: (a) situated Roliball practice targeting a focal technique; (b) high-frame-rate smartphone video capture of selected trials (≥240 fps) and simple kinematic feedback (e.g., racket-head angular velocity) using freely available software; (c) STEAM inquiry tasks prompting students to formulate and test specific, measurable questions; and (d) structured reflection linking empirical observations to conceptual explanations and adjustments to future practice.

A representative inquiry task was: “How does changing the racket angle by 5 degrees affect the landing location in the target zone, and can you use the video to quantify this change?” Students tested two to three preset racket-angle conditions, recorded landing positions across repeated trials, and used frame-by-frame video review to extract simple kinematic/trajectory indicators. Each group summarized findings in a brief worksheet (question–method–results–interpretation) and proposed a technique adjustment for the next practice cycle.

Class time and overall content coverage (e.g., repertoire of Roliball techniques) were matched across groups. Students in the STEAM-RB group primarily used their own smartphones for video capture, with shared tablets or instructor devices as backups when needed.

### 2.5 Measures

The following outcomes were assessed at pretest and posttest: kinematic performance, blinded expert ratings, STEAM knowledge, physical activity motivation, and creative disposition.

#### 2.5.1 Kinematic performance

Kinematic performance was indexed by peak racket-head angular velocity (°/s) during a standardized Roliball forehand drive task at pre- and post-test. Students performed a series of drives toward a marked target zone, while high-frame-rate smartphone video (≥240 fps) was captured in the sagittal plane using student devices or a designated instructor smartphone mounted on a tripod. Video files were transferred to a laptop and analyzed with open-source motion analysis software.

Racket-head angular displacement was tracked frame-by-frame for the forward swing, and angular velocity was derived from the smoothed displacement signal. For each student, three valid trials were analyzed and averaged to yield a single peak angular velocity score at each time point. A random 20% subset of trials was double-coded by two trained analysts; inter-rater reliability for peak angular velocity was excellent, ICC (2, k) = 0.96, 95% CI [0.92, 0.98], consistent with prior work supporting the validity and reliability of smartphone-based kinematic measures in sport tasks.

#### 2.5.2 Blinded expert ratings

Technical quality of the forehand drive was also assessed using blinded expert ratings from standardized video clips. Pre- and post-test clips for each participant were anonymized and randomized. Two experienced Roliball instructors not involved in day-to-day teaching independently rated each clip on a 10-point holistic scale, using a brief rubric emphasizing swing path continuity, segment coordination, timing, and overall control. Raters were blind to group, time point, and study hypotheses. Ratings were averaged to yield a single score (/10) per clip. Inter-rater reliability was excellent, ICC (2, k) = 0.94, 95% CI [0.89, 0.97].

#### 2.5.3 STEAM knowledge

Task-specific STEAM knowledge was assessed through a 15-item criterion-referenced test aligned with the core STEAM concepts embedded in the curriculum (e.g., relationships between racket angle and shuttle trajectory, basic kinematic representations, simple modeling of swing paths). Items included multiple-choice, short-answer, and diagram-based questions, each scored on a 0–3 scale (0 = incorrect, 3 = fully correct), yielding total scores from 0 to 45.

Content validity was evaluated by an expert panel of eight PE and STEAM educators using content validity index (CVI) procedures. Item-level CVIs ranged from 0.88 to 1.00, and the scale-level CVI was 0.94, indicating acceptable to excellent content validity. In the present sample, internal consistency was acceptable (Cronbach’s *α* = 0.85).

### 2.6 Fidelity and implementation checks

To monitor implementation fidelity, instructors completed a brief six-dimension checklist after each class, documenting coverage of key STEAM-RB components (e.g., implementation of all four Kolb-cycle phases, use of smartphone-based feedback and STEAM inquiry tasks), adherence to the traditional syllabus, time allocation to main activities, and notable deviations from the lesson plan. In week 4, the first author observed one session per group using a structured observation form to verify that STEAM-specific elements were implemented only in the STEAM-RB group. No major protocol deviations were identified.

This study does not meet the World Health Organization definition of a clinical trial because the primary outcomes concerned educational and kinematic performance within a routine curriculum context rather than health-related clinical endpoints. The trial was therefore not prospectively registered.

#### 2.6.1 Physical activity motivation

Motivation for physical activity in the PE context was assessed with the Chinese University Students’ Physical Activity Motivation Scale (CUSPAMS), developed and validated under contemporary PE policy constraints. The scale assesses multiple regulatory styles (e.g., intrinsic, identified, introjected, external regulation, amotivation) using 5-point Likert-type items (1 = strongly disagree to 5 = strongly agree). Subscale scores were computed as the mean of constituent items; higher scores reflect stronger endorsement of that regulation.

Given the study focus on autonomous motivation, analyses concentrated on intrinsic and identified regulation indices, which are known correlates of PE engagement and well-being. To provide a more precise test of the SDT-based hypothesis, a composite “autonomous motivation” score was calculated by averaging the intrinsic and identified regulation subscale scores, rather than using a total motivation score that may include controlled regulations. Internal consistency for CUSPAMS subscales in this sample was acceptable to excellent (Cronbach’s *α* = 0.79–0.91).

#### 2.6.2 Creative disposition

Creative disposition was assessed using a revised version of the Creative Personality Scale (CPS) (Gough, 1979) adapted for Chinese university students. The CPS asks respondents to self-rate adjectives reflecting creative tendencies (e.g., imaginative, original) and non-creative attributes, yielding a composite score in which higher values indicate stronger creative disposition. In line with meta-analytic evidence that such traits are relatively stable and slow to change, CPS scores were designated *a priori* as exploratory outcomes. Internal consistency in the present sample was acceptable (Cronbach’s α = 0.76).

### 2.7 Statistical analysis

Analyses were pre-specified around primary, secondary, and exploratory outcomes. Descriptive statistics were computed for all variables. Baseline balance between groups was evaluated using absolute standardized mean differences (ASMDs) (Austin, 2009), with values ≤ 0.15 interpreted as acceptable.

For each continuous outcome, analysis of covariance (ANCOVA) models were fitted with post-test scores as the dependent variable, group (STEAM-RB vs. traditional) as the independent variable, and baseline scores plus prespecified covariates (sex, age, and section) as predictors (European Medicines Agency, 2015). For primary outcomes, adjusted means, adjusted mean differences (STEAM-RB minus traditional), 95% confidence intervals (CIs), *p* values, and partial η^2^ were reported. Secondary and exploratory outcomes were analyzed in analogous models but interpreted cautiously given the number of outcomes and the exploratory status of creative disposition.

Assumptions of ANCOVA (linearity, homogeneity of regression, residual normality and homoscedasticity) were examined and found acceptable. As a robustness check, models were re-estimated with heteroskedasticity-consistent HC3 standard errors; inferences were unchanged. All tests were two-sided with *α* = 0.05. Analyses were conducted in SPSS 26.

## 3 Results

### 3.1 Participant flow and retention

**Figure 2:**
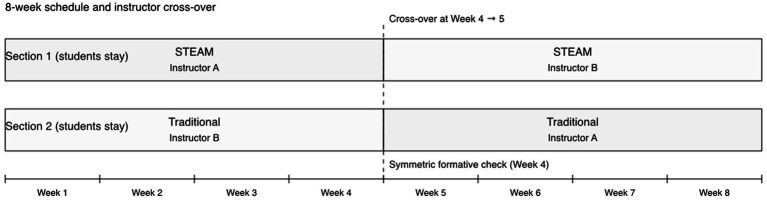
Eight-week modular learning sequence and mid-term instructor crossover. Note. Two intact first-year PE sections received either STEAM-integrated RB or traditional instruction for weeks 1–4, followed by instructor crossover for Weeks 5–8. Time-on-task was matched across sections; contamination was minimized by tool-use restrictions in the traditional condition.

All 70 students who consented were randomized to the STEAM-RB (*n* = 35) or traditional Roliball group (*n* = 35) and completed both pretest and posttest assessments (Figure 2).

Thus, there was no attrition over the 8-week intervention, and all randomized participants were included in the primary analyses. No adverse events related to the intervention were reported.

### 3.2 Baseline characteristics

Baseline characteristics are summarized in Table 1.

**Table 1:** Baseline characteristics with ASMD, t/χ^2^, and *p* (*N* = 70).

Variable	STEAM-RB (*n* = 35)	Traditional (*n* = 35)	ASMD	*t* / χ^2^	*p*
Age (years)	18.5 ± 0.6	18.4 ± 0.7	0.15	0.64 (*df*≈66.4)	= 0.523
Pretest peak racket-head angular velocity (°/s)	128.5 ± 31.0	125.0 ± 29.5	0.11	0.48 (*df*≈67.8)	= 0.630
Pretest blinded expert rating (/10)	2.4 ± 0.8	2.5 ± 0.9	0.12	−0.49 (*df*≈67.1)	= 0.625
BMI (kg/m^2^)	21.6 ± 2.8	21.2 ± 2.5	0.15	0.63 (*df*≈67.1)	= 0.531
Prior sport exposure (years)	5.8 ± 2.1	5.5 ± 2.3	0.13	0.57 (*df*≈67.4)	= 0.571
Weekly physical activity (hours)	3.4 ± 1.5	3.6 ± 1.6	0.13	−0.54 (*df*≈67.7)	= 0.591
Autonomous motivation pretest (score)	3.30 ± 0.58	3.38 ± 0.54	0.14	−0.60 (*df*≈67.8)	= 0.550
STEAM knowledge pretest (score)	15.2 ± 4.8	14.7 ± 5.2	0.10	0.42 (*df*≈67.6)	= 0.677
Sex (male, *n* [%])	21 (60.0%)	19 (54.3%)	0.11	0.23 (*df* = 1)	= 0.629

Values are mean ± SD or n (%). ASMD = absolute standardized mean difference. Welch’s two-sample t-test was used for continuous variables; Pearson’s chi-square test for sex. ASMD ≤ 0.10 indicates excellent balance and ≤ 0.20 acceptable balance. All baseline covariates showed ASMD ≤ 0.15.

The two groups were similar in age (STEAM-RB: 18.5 ± 0.6 years; traditional: 18.4 ± 0.7 years; ASMD = 0.15; *t* ≈ 0.64, *p* = 0.523) and body mass index (21.6 ± 2.8 vs. 21.2 ± 2.5 kg/m^2^; ASMD = 0.15; *p* = 0.531). Pretest peak racket-head angular velocity (128.5 ± 31.0 vs. 125.0 ± 29.5 °/s; ASMD = 0.11; *p* = 0.630) and blinded expert ratings (2.4 ± 0.8 vs. 2.5 ± 0.9/10; ASMD = 0.12; *p* = 0.625) were comparable across groups.

Groups were also balanced on prior sport exposure (5.8 ± 2.1 vs. 5.5 ± 2.3 years; ASMD = 0.13; *p* = 0.571), weekly physical activity (3.4 ± 1.5 vs. 3.6 ± 1.6 h; ASMD = 0.13; *p* = 0.591), and autonomous motivation at pretest (3.30 ± 0.58 vs. 3.38 ± 0.54; ASMD = 0.14; *p* = 0.550). Pretest STEAM knowledge scores were similarly low in both groups (15.2 ± 4.8 vs. 14.7 ± 5.2; ASMD = 0.10; *p* = 0.677). Sex distribution was comparable (21/35 [60.0%] vs. 19/35 [54.3%] male; ASMD = 0.11; χ^2^(1) = 0.23, *p* = 0.629). All baseline covariates showed ASMD ≤ 0.15, indicating acceptable balance after section-stratified randomization.

Before examining group effects, we checked basic measurement properties. Multi-item scales (STEAM knowledge, physical activity motivation, revised CPS) showed acceptable to excellent internal consistency (Cronbach’s *α* ≥ 0.76), and inter-rater reliability for expert ratings and smartphone-derived kinematic variables was in the good-to-excellent range. These indices support the suitability of the measurement battery for detecting curriculum-related changes; detailed coefficients are available on request. Adjusted posttest means and ANCOVA results for all outcomes are presented in Table 2.

**Table 2:** Descriptive statistics and ANCOVA results for post-intervention outcomes (*N* = 70; residual df = 64).

Outcome	Adjusted mean [95% CI] (STEAM-RB)	Adjusted mean [95% CI] (Traditional)	Mean difference (95% CI)	*F*(1, 64)	*p*	partial η^2^
Peak racket-head angular velocity (°/s)	185.0 [175.5, 194.5]	155.0 [145.5, 164.5]	30.0 [16.6, 43.4]	18.53	< 0.001	0.225
Blinded expert rating (/10)	7.5 [7.0, 8.0]	5.5 [5.0, 6.0]	2.0 [1.3, 2.7]	25.41	< 0.001	0.284
STEAM knowledge (score)	45.0 [42.5, 47.5]	20.0 [17.5, 22.5]	25.0 [21.5, 28.5]	50.12	< 0.001	0.439
Autonomous motivation (composite)	4.25 [4.05, 4.45]	3.85 [3.65, 4.05]	0.40 [0.12, 0.68]	7.85	0.007	0.109
Creative disposition (exploratory)	35.2 [32.2, 38.2]	33.0 [30.0, 36.0]	2.2 [−1.5, 5.9]	1.52	0.222	0.023

Adjusted (estimated marginal) means with 95% confidence intervals (CIs) are reported for each outcome. Models adjusted for the corresponding baseline score, age, sex, and class/teacher; statistics include adjusted mean difference (95% CI), F(1, 64), p, and partial η^2^. Creativity was pre-specified as exploratory; effect estimates are presented without confirmatory inference. All tests were two-sided with α = 0.05.

### 3.3 Primary outcomes

#### 3.3.1 Peak racket-head angular velocity

**Figure 3:**
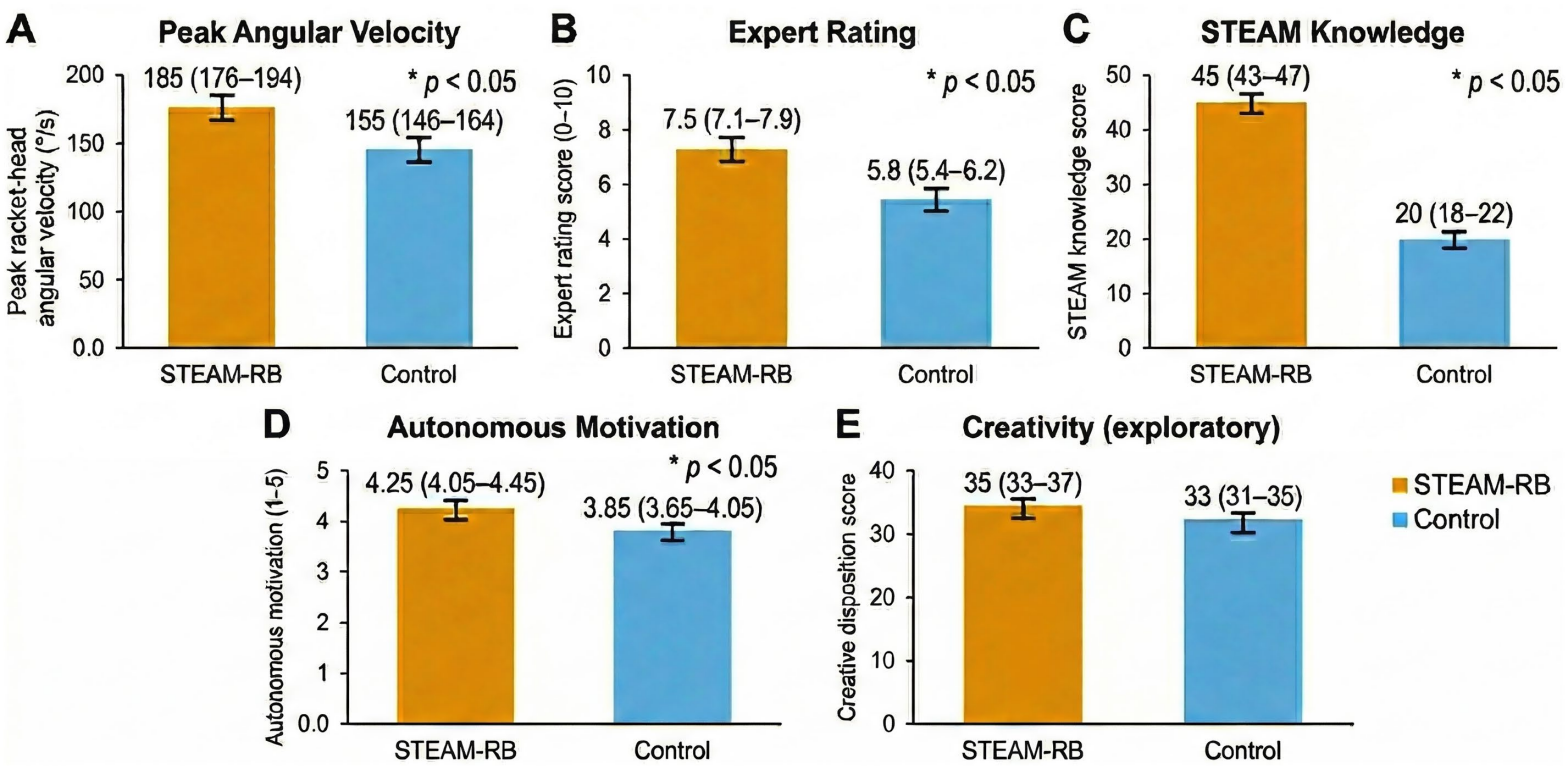
Adjusted post-test outcomes by group (estimated marginal means ± 95% confidence intervals [CIs]). Panels depict ANCOVA-adjusted post-test means for **(A)** peak racket-head angular velocity (°/s), **(B)** blinded expert ratings (0–10), **(C)** STEAM knowledge score, **(D)** autonomous motivation (composite of intrinsic and identified regulation), and **(E)** creative disposition (exploratory outcome). Models were adjusted for the corresponding baseline score, age, sex, and class/teacher. Error bars indicate 95% confidence intervals. Panel-level *F*(1, 64), *p* values, and partial η^2^ are reported in Table 2.

After adjusting for baseline peak racket-head angular velocity, age, sex, and class/teacher, the STEAM-RB group showed substantially higher post-test kinematic performance than the traditional group (Table 2). The adjusted mean peak racket-head angular velocity was 185.0 °/s (95% CI [175.5, 194.5]) in the STEAM-RB group and 155.0 °/s (95% CI [145.5, 164.5]) in the traditional group, yielding an adjusted mean difference of 30.0 °/s (95% CI [16.6, 43.4]). The group effect was statistically significant, *F*(1, 64) = 18.53, *p* < 0.001, with a partial η^2^ of 0.225, indicating a moderate-to-large effect in favor of the STEAM-RB curriculum. Estimated marginal means and 95% CIs are plotted in Figure 3A.

#### 3.3.2 Blinded expert ratings

Blinded expert ratings of forehand drive technique mirrored the kinematic findings. Adjusted post-test expert ratings were 7.5/10 (95% CI [7.0, 8.0]) in the STEAM-RB group and 5.5/10 (95% CI [5.0, 6.0]) in the traditional group (Table 2). The adjusted mean difference was 2.0 points (95% CI [1.3, 2.7]), corresponding to a statistically significant group effect, *F*(1, 64) = 25.41, *p* < 0.001, with partial η^2^ = 0.284. Figure 3B shows the non-overlapping confidence intervals, reinforcing a clear advantage for the STEAM-RB group in perceived technical quality.

#### 3.3.3 STEAM knowledge

The largest primary effect emerged for the STEAM knowledge test. After adjustment, the STEAM-RB group achieved an adjusted mean of 45.0 points (95% CI [42.5, 47.5]) compared with 20.0 points (95% CI [17.5, 22.5]) in the traditional group (Table 2). The adjusted mean difference was 25.0 points (95% CI [21.5, 28.5]), with a highly significant group effect, *F*(1, 64) = 50.12, *p* < 0.001, and a large effect size (partial η^2^ = 0.439). As illustrated in Figure 3C, confidence intervals showed minimal overlap, indicating that the STEAM-RB curriculum produced a substantial gain in task-specific STEAM knowledge relative to the traditional syllabus. Figure 3 summarizes the ANCOVA-adjusted posttest estimated marginal means with 95% confidence intervals for all outcomes.

### 3.4 Secondary outcome: physical activity motivation

For autonomous motivation scores, ANCOVA controlling for baseline autonomous motivation, age, sex, and class/teacher indicated a statistically significant advantage for the STEAM-RB group (Table 2). The adjusted mean post-test motivation score was 4.25 (95% CI [4.05, 4.45]) in the STEAM-RB group versus 3.85 (95% CI[3.65, 4.05]) in the traditional group, yielding an adjusted mean difference of 0.40 points (95% CI [0.12, 0.68]). The group effect was significant, *F*(1, 64) = 7.85, *p* = 0.007, with partial η^2^ = 0.109, indicating a small-to-moderate motivational benefit of the STEAM-RB curriculum.

### 3.5 Exploratory outcome: creative disposition

As an exploratory, trait-like outcome, creative disposition showed limited change over the intervention. Adjusted post-test CPS scores were 35.2 (95% CI [32.2, 38.2]) in the STEAM-RB group and 33.0 (95% CI [30.0, 36.0]) in the traditional group (Table 2). The adjusted mean difference of 2.2 points had a wide confidence interval crossing zero (95% CI [−1.5, 5.9]), and the group effect was not statistically significant, *F*(1, 64) = 1.52, *p* = 0.222, with a small partial η^2^ of 0.023. This pattern is consistent with expectations that broad creative disposition is relatively stable over an 8-week curriculum and may be less sensitive to largely convergent, technique-focused tasks.

Notably, the confidence interval for the group difference (−1.5 to 5.9) cannot rule out a small effect in either direction; however, the primary conclusion remains that the effect was non-significant with a small effect size over the 8-week intervention.

### 3.6 Sensitivity analyses

Sensitivity analyses supported the robustness of these findings. Reduced ANCOVA models adjusting only for baseline scores yielded the same pattern of significance and very similar adjusted mean differences; there was no evidence that section or instructor moderated the curriculum effects.

## 4 Discussion

### 4.1 Summary of principal findings

This section-stratified randomized trial evaluated a technology-enhanced, STEAM-integrated pedagogical framework within a university physical education setting, using Roliball as the intervention context. Compared with a traditional RB syllabus, the STEAM-RB curriculum produced moderate-to-large improvements in peak racket-head angular velocity and blinded expert ratings of forehand technique, and a large gain in task-specific STEAM knowledge. Autonomous motivation (composite of intrinsic and identified regulation) showed a small-to-moderate advantage for the STEAM-RB group, whereas the exploratory outcome of creative disposition changed little and did not differ statistically between groups. Overall, an 8-week, smartphone-supported STEAM-RB curriculum measurably enhanced movement performance, conceptual understanding, and autonomous motivation in university PE, while leaving broad creative personality traits essentially unchanged.

### 4.2 Performance, knowledge, and motivation in a STEAM-integrated PE context

The convergence between kinematic and expert-rated performance provides empirical support for embedding smartphone-based feedback and inquiry tasks within a university PE curriculum. High-frame-rate video and simple kinematic proxies have been shown to yield valid and reliable movement data in applied sport settings (Staacks et al., 2018; Pueo et al., 2020; Mödinger et al., 2022). In this trial, peak racket-head angular velocity distinguished the STEAM-RB and traditional groups with a moderate-to-large effect size, indicating that low-cost smartphone measures were sensitive enough to detect curriculum-level changes over 8 weeks. Blinded expert ratings showed a similar pattern, suggesting that the observed changes are meaningful both instrumentally and from a practitioner’s perspective.

The very large gains in STEAM knowledge are consistent with evidence that STEAM-based instruction can support conceptual understanding when tasks are tightly aligned with disciplinary ideas and embodied practice (Perignat and Katz-Buonincontro, 2019; Tran et al., 2021; Yuan et al., 2022). In the STEAM-RB model, inquiry prompts explicitly connected racket angle, swing path, and timing to ball flight and consistency, inviting students to articulate and test causal explanations rather than simply imitate techniques. The criterion-referenced STEAM knowledge test, developed with documented content validity (Lynn, 1986; Polit and Beck, 2006; Polit et al., 2007), appears well matched to these tasks, yielding a large adjusted mean difference favoring STEAM-RB. From a curricular standpoint, integrating movement practice with targeted STEAM questions and representations can produce substantial cognitive gains in a relatively short university PE unit.

The motivational advantage for the STEAM-RB group was smaller but still meaningful, and was observed specifically for autonomous motivation (the composite of intrinsic and identified regulation). Several curriculum design features aligned with self-determination theory (SDT)—including opportunities to pose and test questions, interpret personalized kinematic feedback, and collaborate on inquiry tasks—likely supported students’ basic psychological needs for competence, autonomy, and relatedness (Ntoumanis et al., 2021; Lin et al., 2022; Wang et al., 2024). For example, smartphone-based feedback may have enhanced perceived competence by making movement technique visible and improvable, inquiry-driven tasks may have strengthened autonomy by allowing students to test their own hypotheses, and collaborative data interpretation may have fostered relatedness through shared problem solving.

At the same time, motivational orientations in PE typically reflect cumulative experiences accrued over many years, making short instructional units more likely to produce incremental shifts rather than wholesale changes in regulatory style. Consequently, larger or more durable gains in autonomous motivation may require longer implementations or program-level adoption of need-supportive, STEAM-integrated pedagogies. This pattern is consistent with SDT-based expectations that brief competence-, autonomy-, and relatedness-supportive interventions tend to produce modest but meaningful improvements in autonomous motivation rather than abrupt changes in overall motivational profiles.

### 4.3 Why creativity did not change

The null findings for creative disposition are consistent with meta-analytic evidence that broad creative personality traits are relatively stable and tend to respond only to long-term or intensive interventions, particularly those involving highly open-ended tasks (Rominger et al., 2022; Vasilopoulos et al., 2023). The revised Creative Personality Scale used here primarily captures trait-like tendencies, which are unlikely to shift substantially in an 8-week PE unit centered on mastering a specific technique. Moreover, the STEAM-RB tasks were predominantly convergent: students refined a “correct” swing path, optimized racket angle, and reduced variability in shuttle trajectory. Although these activities required analytical reasoning, they did not systematically invite divergent ideation or multiple, equally valid solutions. From a measurement perspective, the CPS may therefore have been mismatched to the time scale and openness of the curriculum. Future STEAM-PE studies that aim to influence creativity might employ state-like, task-specific measures (e.g., creative movement tasks, product-based creativity rubrics) and design genuinely open-ended projects, such as student-designed training protocols or equipment modifications, sustained over longer periods.

### 4.4 A transferable blueprint and multi-domain measurement framework

A central contribution of this trial lies in illustrating a multi-domain measurement framework for STEAM-integrated PE that combines kinematic, expert, cognitive, and motivational indicators. Smartphone-based kinematic proxies and blinded expert ratings offered complementary perspectives on movement quality: the former provided fine-grained quantitative sensitivity to change, whereas the latter yielded practitioner-interpretable judgments aligned with typical PE assessment practices. When reliability is established, such low-cost, video-based measures may provide a scalable alternative to laboratory-based motion capture for curriculum evaluation (Pueo et al., 2020; Mödinger et al., 2022).

The STEAM knowledge test showed how curricular tasks can be mapped onto a focused assessment instrument with acceptable content validity, rather than relying on generic science or mathematics tests. Together with the autonomous motivation measure derived from CUSPAMS (Lin et al., 2022) and the exploratory CPS, this battery distinguished outcomes that were highly responsive (knowledge and performance), moderately responsive (motivation), and essentially unresponsive (creative disposition) to the STEAM-RB intervention. Two methodological implications follow. First, outcome domains should be selected to reflect the curriculum’s theory of change, rather than opportunistic use of available instruments. Second, the temporal and conceptual properties of each outcome (state vs. trait, convergent vs. divergent) should be aligned with curriculum duration and task structure.

### 4.5 Implications and transferability beyond Roliball

From a curricular standpoint, the STEAM-RB model shows that integrating smartphone-based kinematic feedback and STEAM inquiry into an existing university PE syllabus is feasible without specialized laboratories or additional contact time. The intervention preserved the core content of the traditional RB curriculum while reconfiguring lesson structures around Kolb’s experiential learning cycle—concrete experience, reflective observation, abstract conceptualization, and active experimentation (Kolb, 2015). Within this cycle, students used their own devices to visualize motion, formulated and tested STEAM-related questions, and iteratively refined technique. In the context of policy calls for PE to contribute both to physical literacy and to broader “future skills” such as problem solving and interdisciplinary thinking (State Council of the People’s Republic of China, 2019; World Economic Forum, 2023), such designs may help reframe university PE as a cognitively rich, inquiry-oriented subject rather than a purely practical requirement.

Although Roliball is a niche, culturally specific sport, the design principles tested here are not unique to RB. Many net-and-wall and implement sports—such as badminton, tennis, pickleball, and emerging racket games—share cyclical stroke patterns, ball–racket interactions, and trajectory constraints that can be visualized through smartphone video and simple kinematic proxies (Cooke, 1999; Haake et al., 2003; Pueo et al., 2020). Work using inertial measurement units and gyroscopes to estimate angular velocity during tennis strokes further demonstrates that relatively inexpensive sensors can capture meaningful aspects of racket dynamics in applied settings (Delgado-García et al., 2021; Brocherie and Dinu, 2022). The STEAM-RB model thus offers a transferable template: identify a “signature” technique, map it onto a small set of STEAM questions, and embed low-cost sensing within an experiential learning cycle supported by guided inquiry. Future adaptations could test analogous modules in badminton, tennis, or pickleball, preserving the data-informed inquiry structure while drawing on locally meaningful cultural narratives (Steyn and Rix, 2025).

### 4.6 Strengths, limitations, and future directions

Strengths of this trial include the section-stratified individual randomization with an instructor crossover, covariate-adjusted ANCOVA models, implementation under authentic university PE conditions, and a multi-domain measurement framework that combined instrumented and expert-rated performance, STEAM knowledge, and motivation. These features distinguish the study from typical quasi-experimental PE research and support cautious causal inference.

Several limitations should also be acknowledged. The trial was conducted at a single public university with a modest sample (*N* = 70) and two instructors, limiting generalizability to other institutions and populations. The 8-week duration precluded examination of long-term maintenance and may have been too short to affect more stable constructs such as creative disposition. Smartphone hardware and software varied across students; however, contemporary smartphone cameras generally provide sufficiently high frame rates and image quality for movement analysis in applied settings. Reliability checks supported the kinematic measures used in this study, and a brief post-hoc examination did not suggest systematic differences in outcomes associated with device source (student smartphones vs. instructor/backup devices). Nevertheless, systematic device calibration and direct comparisons with gold-standard motion capture systems were beyond the scope of this trial. Future research could more formally evaluate whether device specifications (e.g., operating system, camera frame rate, or sensor characteristics) moderate measurement precision or intervention effects. Finally, the STEAM knowledge test was tightly aligned to RB-specific content, so scores should not be interpreted as reflecting general STEAM competence, and the sample comprised first-year Chinese undergraduates, which may limit transferability to other age groups or education systems.

Future research could extend this work through multi-site trials across different types of universities and regions, longer interventions spanning a semester or year, and more intensive validation of smartphone-based kinematic measures against laboratory systems and inertial sensors. Designing STEAM-PE units that culminate in student-generated training plans, equipment innovations, or game modifications, coupled with state-like creativity measures and qualitative work on students’ experiences as “scientific movers,” may clarify how STEAM-integrated PE can contribute not only to performance, knowledge, and motivation, but also to creative engagement with movement.

## 5 Conclusion

This randomized trial shows that a STEAM-integrated Roliball curriculum, grounded in experiential learning and supported by smartphone-based kinematic feedback, can produce meaningful improvements in movement performance, STEAM knowledge, and overall motivation within a university PE course. By demonstrating how a multi-domain measurement framework can be implemented with low-cost tools in an authentic PE setting, the study contributes to both the design of STEAM-integrated PE curricula and the methodological toolkit available for their evaluation. The limited effects on creative disposition highlight the importance of aligning outcome choice with curriculum duration and task openness. Thoughtfully designed STEAM-PE units may help universities move toward physically, cognitively, and motivationally richer PE experiences, while also clarifying the measurement and design challenges that remain.

## Data Availability

The raw data supporting the conclusions of this article will be made available by the authors, without undue reservation.
